# Case Report: Transformation From Non-Small Cell Lung Cancer to Small Cell Lung Cancer During Anti-PD-1 Therapy: A Report of Two Cases

**DOI:** 10.3389/fonc.2021.619371

**Published:** 2021-05-21

**Authors:** Qian Shen, Jingjing Qu, Lingyan Sheng, Qiqi Gao, Jianying Zhou

**Affiliations:** ^1^Department of Respiratory Disease, Thoracic Disease Centre, The First Affiliated Hospital, College of Medicine, Zhejiang University, Hangzhou, China; ^2^Department of Pathology, The First Affiliated Hospital, College of Medicine, Zhejiang University, Hangzhou, China

**Keywords:** transformation, small-cell lung cancer, lung squamous cell carcinomas, PD-1 inhibitors, resistant

## Abstract

**Background:**

Histological transformation of lung cancer to small cell lung cancer (SCLC) is uncommon. It is a small subset of the possible resistance mechanisms, even in epidermal growth factor receptor (EGFR)-mutated non-small cell lung cancer treated with EGFR-tyrosine kinase inhibitors. Reports on programmed cell death-1 (PD-1) inhibitors are rare. We report two cases of lung squamous carcinomas that transformed to SCLC during anti-PD-1 therapy, and present a detailed description of histological examination of the pre-and post-transformation tissues, hitherto absent from reports on the topic.

**Case Presentation:**

Case 1: A 69-year-old man was diagnosed with stage IVa squamous cell carcinoma of the lung. He had a programmed cell death-ligand 1 tumor proportion score ≥50%. He achieved partial response after four cycles of sintilimab as first-line treatment. However, sintilimab was discontinued because of severe decrease in hemoglobin levels and platelet counts. Moreover, the occurrence of pleural effusion favored disease progression. Interestingly, bone marrow puncture and biopsy showed transformation to SCLC. Case 2: A 71-year-old man diagnosed with stage IIIa lung squamous cell carcinoma received neoadjuvant chemotherapy, underwent radical surgery, and finally received adjuvant chemotherapy. Five months later, he presented with tumor recurrence. He was treated with nivolumab, though disease progression was observed after four cycles. Notably, a subsequent computed tomography-guided biopsy showed SCLC.

**Conclusion:**

Phenotypic transformation to SCLC is a potential mechanism of resistance to immunotherapy in squamous cell carcinomas of the lung. Disease progression should prompt re-biopsy to diagnose potential histological changes to assess the requirement for change in treatment.

## Introduction

Lung cancer remains the leading cause of tumor-related deaths worldwide. Its incidence has increased steadily by 10% annually (1–3). It can be divided into two histological subtypes: non-small cell lung cancer (NSCLC) and small cell lung cancer (SCLC). NSCLC accounts for approximately 85% of lung cancers, and includes adenocarcinoma, squamous cell carcinoma, adenosquamous carcinoma, and large cell carcinoma; SCLC accounts for 15% (4). Recently, targeted therapies based on driver genes and immune checkpoint inhibitors (ICIs) have led to a significant improvement in patient survival. ICIs are the mainstay of treatment for lung cancer (5). Programmed cell death 1 (PD-1)/programmed cell death-ligand 1 (PD-L1) inhibitors are the standard first-line and second-line therapies for patients with NSCLC without driver gene mutations (6, 7). Sintilimab is an anti-PD-1 antibody that was co-developed by Innovent Biologics and Eli Lilly and Company. A combination of sintilimab and chemotherapy has been shown to result in a longer progression-free survival than chemotherapy alone in patients with untreated, locally advanced or metastatic NSCLC (8). However, the development of resistance to PD-1/PD-L1 inhibitors is one of the challenges in cancer treatment. It is well known that histological transformation to SCLC is a known mechanism of resistance in 3–14% of patients with epidermal growth factor receptor (EGFR) mutations receiving EGFR tyrosine kinase inhibitors (EGFR-TKIs) (9–11). Similarly, transformation of NSCLC to SCLC in patients receiving PD-1/PD-L1 inhibitors has been reported as a mechanism of resistance to PD-1/PD-L1 inhibitors (12–15). Unlike with EGFR-TKI therapy, repeat tissue biopsies are not usually performed at the time of NSCLC progression in patients treated with PD-1/PD-L1 inhibitors. Herein, we report two cases with squamous cell carcinoma that transformed into SCLC during treatment with PD-1 inhibitors. We also provide a detailed description of the histological examination of the pre-and post-transformation tissues, hitherto absent from prior reports (12–15).

## Case Description

### Case 1

**Figure 1:**
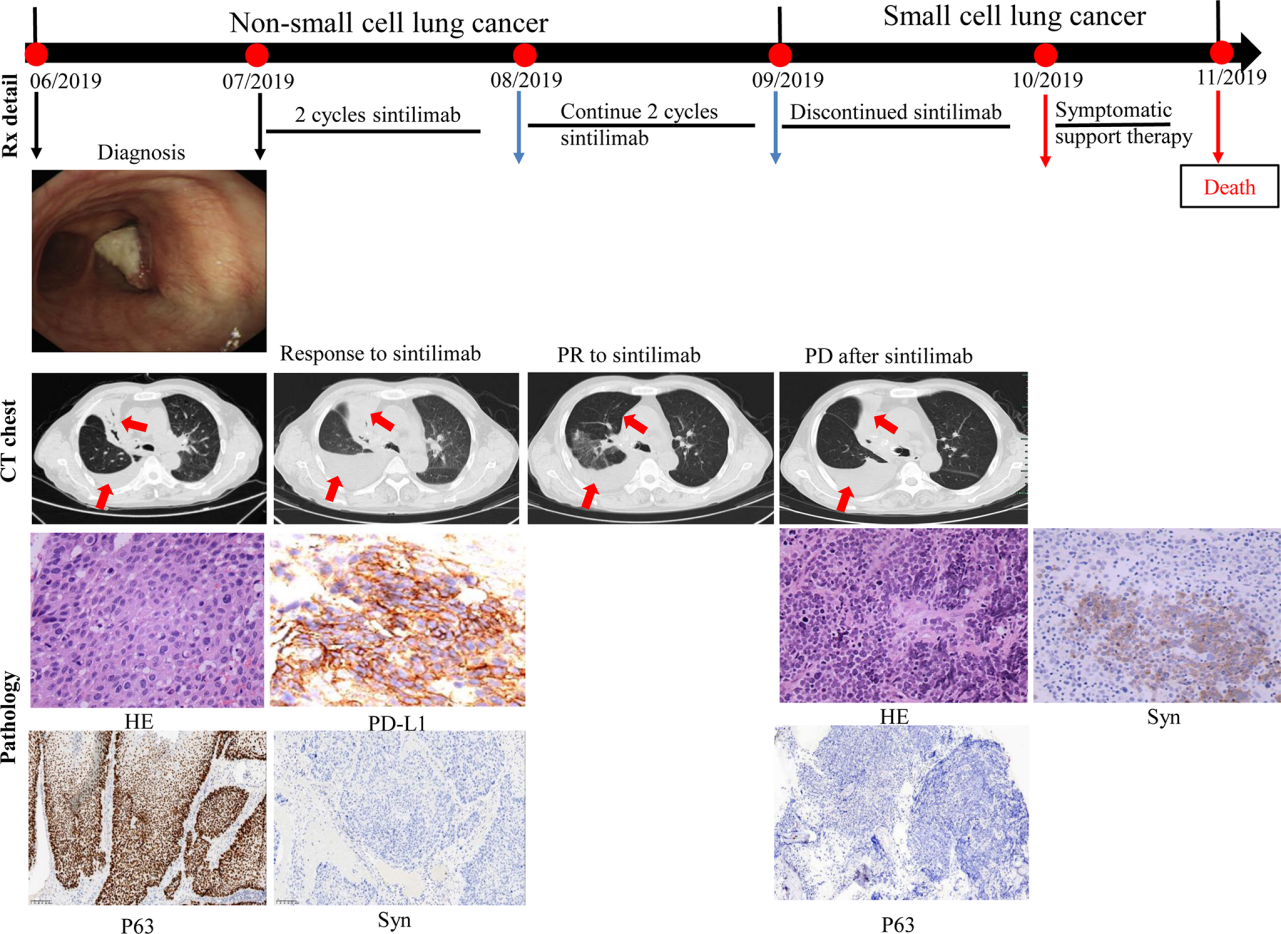
Case presentation of transformation of lung squamous cell carcinoma to small cell lung carcinoma with first-line sintilimab monotherapy. CT, computed tomography; HE, Hematoxylin and Eosin; Syn, synaptophysin; Rx, treatment.

A 69-year-old Chinese man with a 30-pack year smoking history presented with cough and decreased hemoglobin levels and platelet counts. The patient had a history of myelodysplastic syndrome and chronic obstructive pulmonary disease. Chest computed tomography (CT) performed in June 2019 showed right upper central lung cancer with right upper obstructive atelectasis, right hilar and mediastinal lymph node enlargement, and mild right-sided pleural effusion. No metastasis was observed on magnetic resonance imaging (MRI) of the brain and emission computed tomography (ECT) of the bone. Serum levels of the tumor markers: carcinoembryonic antigen (CEA) and neuron-specific enolase (NSE) were within normal range. Fiberoptic bronchoscopy revealed a new biological blockage in the right main bronchus. The patient underwent neoplasm excision and stent implantation in the right main bronchus. Finally, the patient was diagnosed with stage IVa squamous cell carcinoma of the lung with a PD-L1 tumor proportion score (TPS) ≥50%. He received sintilimab, a PD-1 inhibitor, as the first-line treatment in July 2019 for PD-L1 overexpression. After two cycles of sintilimab therapy, CT showed a massive right-sided pleural effusion, which increased significantly in August 2019. However, the patient did not have any clinical symptoms such as fever or cough. Hence, two more cycles of sintilimab were administered. A follow-up CT was performed in September 2019; it showed that the patient had achieved partial response, and the right lung atelectasis and right-sided pleural effusion had regressed. However, the patient developed systemic edema, and experienced huge decrease in hemoglobin levels and platelet counts; CT showed that the right lung mass and obstructive atelectasis of the right lung had progressed at October 2019. Moreover, NSE levels were high (302.3 ng/ml, normal range 0.0–30.0 ng/ml). A bone marrow biopsy showed tumor cells that were positive for synaptophysin (Syn), chromogranin A (CgA), and CD45, suggestive of SCLC. The patient died of respiratory failure, severe anemia, and severe thrombocytopenia (**Figure 1**).

### Case 2

**Figure 2:**
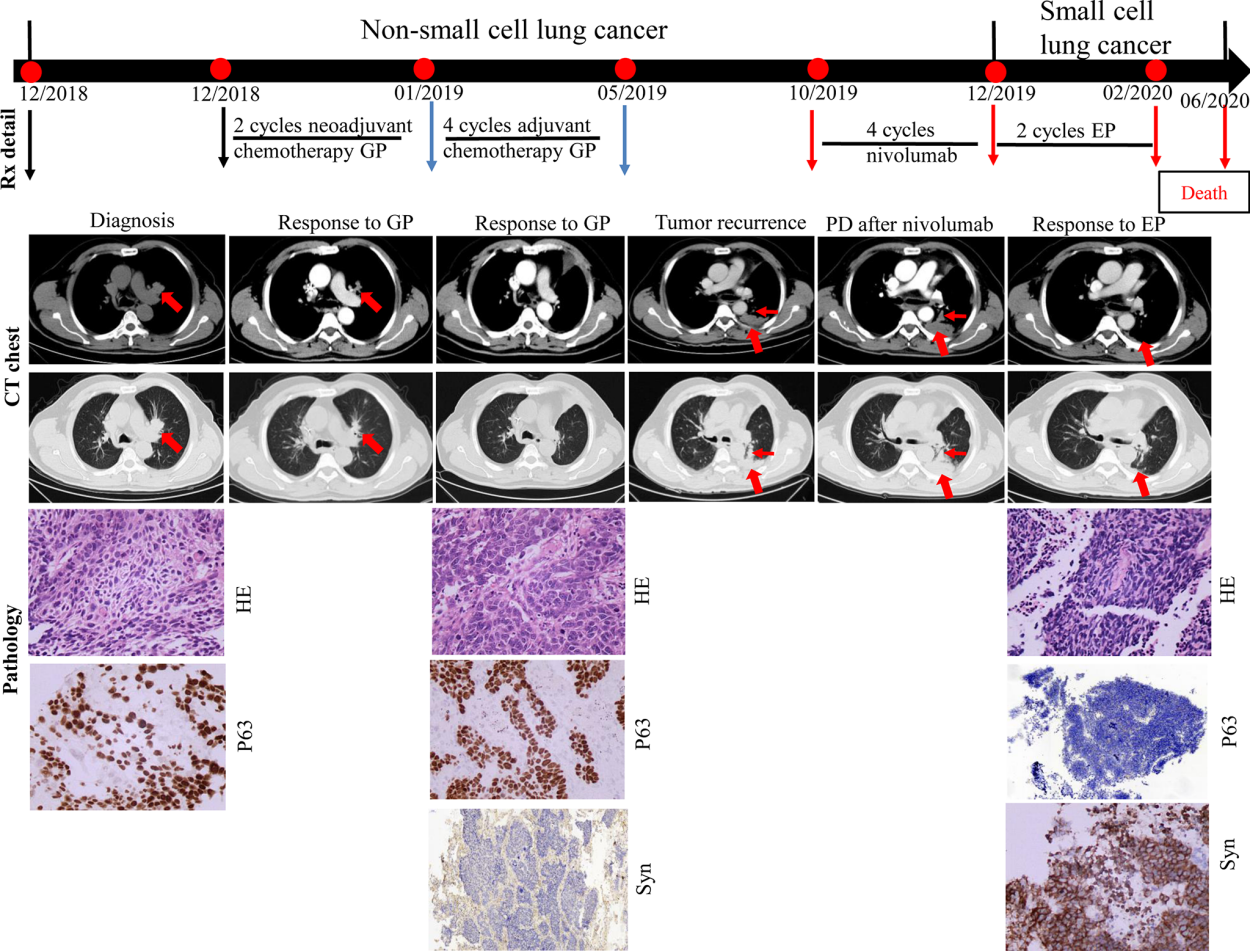
Case presentation of transformation of lung squamous cell carcinomas to small cell lung carcinoma with nivolumab. CT, computed tomography; HE, Hematoxylin and Eosin; IHC, immunohistochemical; G, gemcitabine; P, cisplatin; E, etoposide; Syn, synaptophysin; Rx, treatment.

A 71-year-old man presented with productive cough. A CT scan confirmed the presence of an irregular mass in the left upper lobe. Positron emission tomography confirmed the presence of mediastinal lymph nodes. Serum CEA and NSE levels were normal. Fiberoptic bronchoscopy revealed a neoplasm at the opening of the upper branch of the left bronchus. A diagnosis of stage IIIa squamous cell carcinoma of the lung (cT1cN2M0) was made in December 2018 based on bronchoscopy-guided biopsy results (P63 positive). Two cycles of neoadjuvant chemotherapy with cisplatin-gemcitabine were administered. A CT scan performed in January 2019 showed that the mass in the left upper lobe had reduced significantly. Therefore, the patient underwent radical surgery of the left upper lung. Pathological biopsy of the surgical specimen revealed medium-poorly differentiated squamous cell carcinoma with par-bronchogenic lymph node metastases (pT1cN1M0, IIb); the tumor was P63-positive and Syn-negative. Postoperatively, the patient received four cycles of adjuvant chemotherapy with cisplatin-gemcitabine until May 2019. Five months after adjuvant chemotherapy, the patient developed tumor recurrence in the left lung with left pleural metastases. A combination of PD-1 inhibitors and docetaxel was prescribed based on the PD-L1 TPS expression of 1% (22C3 test). However, he refused docetaxel therapy. After two additional months of nivolumab monotherapy (four cycles), the CT scan showed tumor progression and metastases in the left lung and left pleura, compared to the CT findings of October 2019. Further, NSE levels were high (92.3 ng/ml). A CT-guided biopsy of the left pleural mass revealed that the tumor cells were positive for Syn and negative for P63; these findings were consistent with SCLC. From December 2019, he received two cycles of cisplatin and etoposide chemotherapy. The size of metastases in the left lung and left pleura decreased significantly, and the NSE levels decreased to 14.9 ng/ml. However, bone ECT showed metastases in the seventh, ninth, and twelfth thoracic vertebrae. The patient was thus diagnosed with disease progression. A combination of chemotherapy and internal radiotherapy with Strontium-89 was recommended to the patient, but he declined any further treatment. Finally, he died of the primary disease during the fourth month of follow-up (**Figure 2**).

Detailed clinicopathological characteristics of the two patients are summarized in **Table 1**.

**Table 1:** Summary of clinicopathological characteristics of the two cases.

Case No	Age	Sex	Smoking history, years	Histology	PD-L1 expression	Treatment of ICIs	Best response to ICIs	Treatment for SCLC	Patients outcome post SCLC therapy
1	69	Male	30	SCC	≥50%	Sintilimab (q3wk)	PR	NA	Died
2	71	Male	34	SCC	1%	Nivolumab (q2wk)	PD	EP	Died

SCC, squamous cell carcinomas; PR, partial response; PD, progression of disease; EP, etoposide plus cisplatin; ICIs, immune checkpoint inhibitors; PD-L1, programmed cell death-ligand 1.

## Discussion

There is no consensus on the definition of NSCLC-to-SCLC transformation and its differences from primary SCLC. In our two patients, the initial biopsy showed no neuroendocrine features, and histological findings in favor of SCLC were found on re-biopsy, which was prompted by radiological signs of disease progression. Two independent pathologists reviewed the pathological sections. The histological transformation of lung adenocarcinoma to SCLC has been found in 3–14% of patients treated with EGFR-TKIs (9–11). However, there are only a few published cases of histological transformation to SCLC in patients receiving PD-1 inhibitors, and retrospective studies are lacking. Sehgal and Imakita reported disease progression in a patient who received nivolumab monotherapy as second-line therapy; re-biopsy showed transformation to SCLC (12, 16). lams reported that transformation to SCLC could be a mechanism of resistance to nivolumab in KRAS-mutant lung adenocarcinoma (15). Similarly, histological transformation to SCLC has also been reported with pembrolizumab therapy (17).

Transformation to small-cell carcinoma is a known mechanism of resistance to EGFR-TKIs in adenocarcinoma. One possible explanation is the low sensitivity to treatment for a small number of tumor cells, and the higher proportion of the small-cell carcinoma component in the initial tumor due to selective pressure. Recently, studies revealed that transformation to SCLC was associated with inactivation of Retinoblastoma 1 and p53, which promote small-cell cloning from adenocarcinoma at an early stage due to divergent evolutionary processes (18). Transformation to small-cell carcinoma may also be a mechanism of resistance to PD-1 inhibitors in NSCLC (13). In our patients, rapid tumor progression was observed after only four cycles of ICIs, despite PD-L1 expression in the tumor tissue. We should take into consideration that the original tumors in our two patients were probably a combination of small-cell carcinoma and squamous cell carcinoma, which were not detected in the initial biopsy specimen, explained by the low SCLC component that was not detectable in the initial transbronchial lung biopsy sample. The other possibility is that the NSCLC cells underwent histological transformation to SCLC cells due to the PD-1 inhibitor. However, while the mechanism is unclear, this possibility is supported by previous studies. In previous reports of transformation to SCLC in EGFR-mutant adenocarcinoma, the EGFR mutation was identical in all the transformed SCLC tissue (19, 20). The SCLC-transformation cases described in our series may be isolated examples of a general phenomenon of resistance to ICIs due to a loss of mutations associated with neoantigens or other tumor cell-dependent elements of the immune response. From the clinical trials performed so far, the efficacy of ICIs may be lower in SCLC than in NSCLC, which is possibly supportive of transformation to SCLC as a mechanism of escape from immunotherapy (21). Furthermore, pathology and immunohistochemistry are the gold standard for SCLC diagnosis. Next-generation sequencing (NGS) is a technology used by many laboratories to test for inherited disorders and tumor mutations. However, given that the rate of gene mutation in SCLC is relatively low, we did not consider NGS as an option for our patients. Nonetheless, in the best-case scenario, NGS and molecular fingerprinting would be the next best step and would help in determining the origin of SCLC and may also help elucidate the mechanism of resistance.

In conclusion, we reported two cases of squamous cell carcinoma of the lung, which transformed to SCLC during immunotherapy with PD-1 inhibitors. Phenotypic transformation to SCLC is a potential mechanism of resistance to immunotherapy in squamous cell carcinomas of the lung. Clinicians should be aware of this transformation and consider re-biopsy after PD-1 therapy for NSCLC, especially in patients with disease progression.

## Data Availability Statement

The original contributions presented in the study are included in the article/supplementary material. Further inquiries can be directed to the corresponding author.

## Ethics Statement

Written informed consent was obtained from the individual(s) for the publication of any potentially identifiable images or data included in this article.

## Author Contributions

QS and JZ provided patient information. JQ, QG, and QS collected the data. QS and JZ were responsible for study conception and design and acquiring financial support. QG reviewed the pathological sections and took pathological photos. LS critically revised the manuscript for intellectual content. All authors contributed to the article and approved the submitted version.

## Funding

This work was supported by grants from the Medicine and Health Project of Zhejiang Province, China (Grant No. 2018KY061).

## Conflict of Interest

The authors declare that the research was conducted in the absence of any commercial or financial relationships that could be construed as a potential conflict of interest.
